# Mechanistic two‐pathway modeling of substrate inhibition in lactic acid bacteria for enhanced fermentation control

**DOI:** 10.1002/qub2.70019

**Published:** 2025-10-06

**Authors:** Guoxi Zheng, Junwen Mao

**Affiliations:** ^1^ College of Science Huzhou University Huzhou Zhejiang China; ^2^ School of Physics and Astronomy Yunnan University Kunming Yunnan China

**Keywords:** inoculum size, lactic acid bacteria, lag phase, physiological state, substrate inhibition

## Abstract

Substrate inhibition in lactic acid bacteria (LAB) fermentation occurs when substrate concentration exceeds a critical value, leading to reduced cell growth and thus inefficient lactic acid production. Many efforts, including experimental and kinetic models, have been devoted to elucidate the possible mechanisms of substrate inhibition. However, the molecular and physiological basis of this phenomenon remains incompletely characterized. In this study, we propose a mechanistic two‐pathway model that integrates a substrate‐responsive molecular regulatory pathway into the typical substrate assimilation and microbial growth pathway. Our modeling analysis captures a global growth dynamics, including lag, exponential, and stationary phases over a wide range of initial substrate concentrations, with one set of parameters. Consequently, the results exhibit a significantly prolonged lag phase at high initial substrate concentrations. We test this model framework by combining the model results with the published experimental data of LAB batch fermentation such as *Lactobacillus bulgaricus*, *Lactobacillus casei*, and *Lactiplantibacillus plantarum* on lactose, demonstrating its universality beyond specific substrate‐strain systems. Furthermore, the model simulations show that an appropriate preculture treatment for modulating the inoculum’s physiological state of the population could be a possible approach to cope with the challenge of substrate inhibition at high‐substrate environments. Finally, the model predictions of optimal microbial growth dynamics are investigated from various inoculum sizes. The proposed modeling approach provides novel insights into the connection between microbial fermentation and substrate supply, facilitating efficient substrate utilization in bioprocess engineering.

## 1 INTRODUCTION

Over recent decades, the lactic acid (LA) production has progressively shifted toward optimized fermentation processes using agro‐industrial wastes as carbon sources, driven by their low‐cost and high‐efficiency substrate conversion [1, 2, 3, 4]. In these bioprocesses, the fermentable carbohydrates are hydrolyzed into monosaccharides (e.g., glucose and galactose) for further metabolism. However, a persistent challenge of substrate inhibition hinders microbial growth and thus leads to a concomitant decline of LA productivity under high substrate conditions [5, 6].

To elucidate substrate inhibition in microbial growth, lots of works have been done to attempt to employ a straightforward relation between the specific growth rate of microbial growth and substrate concentration [5, 6, 7, 8, 9, 10]. For instance, the Haldane–Andrews equation [9, 10] was often used to describe substrate inhibition of microbial growth [7]. Another frequently used inhibition function is the Aiba–Edwards equation [7, 11], which considered the inhibition of growth as an effect of product inhibition. Although these modified Monod equations well reproduced the experimental data of suppressive growth in many fermentation processes by statistical curve fitting [5, 10, 12], they were still far from elucidating the underlying mechanisms of substrate inhibition, and the wide variability of fitting curves arising from different inhibition functions usually made it hard to assess these empirical functions when only statistical techniques were involved [10, 13, 14]. Indeed, most of these studies focused on a specific bioprocess, aiming at providing optimal parameters for the design of industrial bioprocesses. As a consequence, the expressions of these models varied widely with biological processes and microorganism species [3, 5].

It is well known that a typical bacterial growth curve usually shows lag phase, exponential phase, stationary phase, and sometimes death phase under nutrient‐limited conditions [15, 16]. However, the importance of lag phase is substantially undervalued as compared to other bacterial growth phases, both experimentally and theoretically [17, 18]. In fact, despite no distinct growth being observed in the lag phase, increasing evidence suggests that it is metabolically active in response to environmental stress [19, 20, 21, 22]. For example, it has been reported that accumulation of sugars during enzymatic hydrolysis of lignocellulose was accompanied by the inhibition of enzymatic activity, leading to a prolonged lag time in batch fermentation, and thus reducing the average productivity of LA [23]. However, it is still unclear how the physiological process in lag phase works at various substrate concentrations, and how nutrient supply affects microbial growth dynamics as well as the bacterial community structure. Therefore, an integrative framework combining quantitative experimentation with mathematical modeling is indispensable to providing important clues for understanding microbial growth in harsh environments and optimizing substrate utilization in industry bioreactors [18].

Microbial fermentation is a complex process that is largely dictated by substrate supply and utilization [24, 25, 26, 27]. It has been observed experimentally that several enzymes, such as lactose permease and β‐galactosidase, play a crucial role in hydrolysis of carbon substrates [28, 29]. A recent study on lactose utilization of *Lactiplantibacillus plantarum* showed that cells will not be able to grow if lacking β‐galactosidase in the environments where lactose is the only substrate [30]. Meanwhile, the synthesis of these enzymes can be significantly affected by substrate concentration. Prior experiments have observed that the activity of β‐galactosidase was possibly inhibited by a high concentration of metabolic intermediates arising from substrate metabolism [31, 32, 33], leading to an inefficient substrate utilization and reduced microbial growth. Hence, the rational regulation of the activity of these enzymes is important to optimize cell growth and product synthesis in microbial fermentation.

This work aims to investigate the kinetics of substrate inhibition in lactic acid bacteria (LAB) fermentation under batch fermentation conditions, with a focus on a physiologically adaptive response to different substrate concentrations. Based on the experimental data of fermentation processes from diverse microorganisms, the predictive modeling substrate inhibition was evaluated.

## 2 MODELS AND RESULTS

### 2.1 The Monod equation

Under circumstances where resources are limited, the microbial growth rate can be well described by the Monod model [34], which correlates specific growth rate (μ) with substrate concentration through a monotonically increasing function. The rate of change of microbial population reads as follows in Equation (1),

(1) $$\frac{dX}{dt}=\mu_{\max}\frac{S}{K_{S}+S}X$$

where X denotes the concentration of the bacteria, S is the substrate concentration; $\mu_{\max}$ is the maximum growth rate for $S\gg K_{S}$, and $K_{S}$ is the Monod constant. The Monod growth $\mu_{S}=\frac{S}{K_{S}+S}$ indicates a monotonically increasing growth rate of bacteria with the growing concentration of substrate.

### 2.2 The Baranyi–Roberts model

To characterize the lag phase in microbial growth kinetics, Baranyi and Roberts [35] proposed a predictive model by postulating an intracellular component that is responsible for regulating cell growth as the critical substance. The model can be described by Equation (2),

(2) $$\frac{dX}{dt}=\mu_{\max}\cdot \mu_{Q}\left(t\right)\cdot \frac{S}{K_{S}+S}\cdot X$$

where $\mu_{Q}\left(t\right)$ is a time‐varying adjustment function ($0\leq \mu_{Q}\left(t\right)\leq 1$), which describes the degree of adaptability of the population to a new growth condition [35, 36]. Following assumptions of Baranyi and Roberts, the adjustment function can be written as Equation (3),

(3) $$\mu_{Q}=\frac{Q\left(t\right)}{1+Q\left(t\right)}$$

where Q(t) represents the level of physiological state, assumed to be proportional to the concentration of certain critical substance in a cell [35]. The Baranyi–Roberts model, Equations (2) and (3), enable the characteristic of the duration of lag time via changing physiological states of the cells. Particularly the physiological state of the inoculums $Q_{0}$, relevant to growth in the previous medium, can affect the adaptation to a new medium [36, 37].

### 2.3 Two pathway model of substrate utilization

**FIGURE 1:**
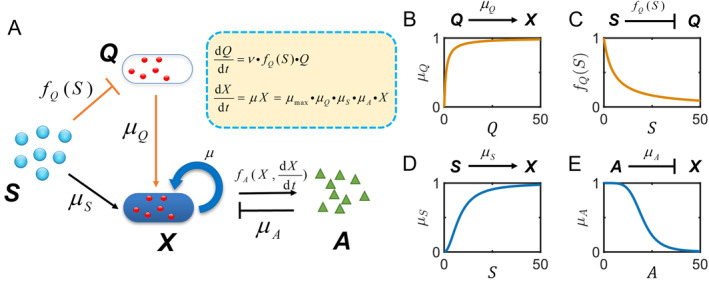
Two pathway modeling substrate inhibition kinetics in lactic acid bacteria fermentation. (A) Schematic framework of the two‐pathway model. Inset box: the mathematical equations of protein synthesis and microbial growth. (B) The adjustment function $\mu_{Q}$. (C) The inhibition function $f_{Q}\left(S\right)$. (D) The growth rate $\mu_{S}$. (E) The product inhibition factor $\mu_{A}$.

In this work, we propose an integrated coarse‐grained model which consists of two pathways for substrate utilization: microbial growth pathway (bottom) and the substrate‐responsive regulatory pathway (upper), as shown in Figure 1A. The typical growth pathway describes the conversion of substrate *S* to the growth of bacteria *X* and subsequent production of *A*. As the fermentation product LA may in turn have inhibitory effects on bacterial growth, product inhibition is also included. In the substrate‐responsive regulatory pathway, an intracellular protein *Q* is introduced as the critical enzyme to regulate substrate utilization and cell growth.

Here, microbial growth is characterized to be subject to the adjustment function as shown in Equation (3), in which the physiological state of the cells is assumed to be dependent on the intracellular protein *Q*, as shown in Figure 1B. In fact, it is a challenge to describe cell growth dynamics in terms of a specific critical enzyme responsible for regulating the physiological state because often a group of enzymes with diverse functions are involved in facilitation of substrate metabolism. For example, as mentioned above, several proteins such as lactose permease and β‐galactosidase work in concert with each other in lactose metabolism [28, 29]. Recent experiments have observed that the expression of lactose hydrolyzing enzyme β‐galactosidase catalyzed the conversion of lactose into glucose and galactose for further metabolism, enabling substrate utilization for cell growth and energy maintenance [29, 38]. Therefore, the modeling approach keeps the main characteristic of these enzymes as the critical substance at the coarse‐grained level while making the model mathematically tractable.

Under the assumption of the Baranyi–Roberts model, the time‐dependent level of the physiological state follows a first‐order kinetics: $\frac{dQ}{dt}=\nu Q$, where ν measures how fast the population adapts to a new environment [35]. This means that the time‐dependent physiological state is usually modeled by an exponential evolution of Q(t), with $Q_{0}$ as the initial physiological state.

In fact, lots of previous experimental observations on production of β‐galactosidase have reported that the enzyme synthesis was likely suppressed by a consistent accumulation of substrate [31, 32, 33]. Here, we hypothesize that the synthesis of critical protein is limited to the increasing substrate supply, following a Hill function $f_{Q}\left(S\right)=\frac{1}{1+{\left(S/K_{Q}\right)}^{n_{Q}}}$ (Figure 1C). Therefore, the evolution of the physiological state Q can be written as Equation (4),

(4) $$\frac{dQ}{dt}=\nu \frac{1}{1+{\left(S/K_{Q}\right)}^{n_{Q}}}Q$$

where $K_{Q}$ is the substrate inhibition constant; and $n_{Q}$ is model parameter. In this work, we take $K_{Q}=K_{S}$ for simplicity. The rate ν characterizes the quickness of adaptation to the new environment. Following the Baranyi and Roberts model, the rate is assumed to be same as maximum specific growth rate, $\nu =\mu_{\max}$ for bottle‐neck growth, as was done in Ref. [35].

As shown in Figure 1D, the Monod growth $\mu_{S}=\frac{S}{K_{S}+S}$ is taken to model the typical microbial growth, as shown in Equation (1). In addition, the factor $\mu_{A}=\frac{1}{1+{\left(A/K_{I}\right)}^{n_{A}}}$ is the noncompetitive inhibition arising from the fermentation product A (Figure 1E), where $K_{I}$ is the product inhibition constant; and $n_{A}$ is model parameter.

The production of LA is described by the Luedeking–Piret equation as shown in Equation (5) [39, 40],

(5) $$f_{A}\left(X,\frac{dX}{dt}\right)=\alpha \frac{dX}{dt}+\beta X$$

where X is the concentration of bacteria, and the parameter α and β are constants for growth‐ and nongrowth‐associated LA production, respectively.

### 2.4 Integrated modeling of substrate inhibition

To summarize, in this work we propose an integrated two pathway model to characterize microbial growth kinetics of substrate inhibition. The overall growth rate μ of the microorganism is subject to four growth factors which can be written as Equation (6),

(6) $$\mu =\mu_{\max}\mu_{Q}\mu_{S}\mu_{A}$$

where $\mu_{\max}$ is the maximum growth rate that is a constant for a strain. As discussed above, $\mu_{Q}$ is the adjustment function of microbial growth due to the accumulation of the critical protein, $\mu_{S}$ measures substrate conversion to cell growth in form of the Monod growth kinetics, and $\mu_{A}$ describes product inhibition from fermentation product *A*.

In terms of Equations ((3), (4), (5))–(6), the set of differential equations of microbial fermentation process of substrate utilization, physiological state evolution, biomass growth and LA formation can be described as Equation (7),

(7) $$\begin{array}{l} \frac{dS}{dt}=-\frac{1}{\gamma_{X/S}}\frac{dX}{dt}-\frac{1}{\gamma_{A/S}}\frac{dA}{dt}\\ \frac{dQ}{dt}=\nu \frac{1}{1+{\left(S/K_{Q}\right)}^{n_{Q}}}Q\\ \frac{dX}{dt}=\mu X=\mu_{\max}\frac{Q\left(t\right)}{1+Q\left(t\right)}\frac{S/K_{S}}{1+S/K_{S}}\frac{1}{1+{\left(A/K_{I}\right)}^{n_{A}}}X\\ \frac{dA}{dt}=\alpha \frac{dX}{dt}+\beta X \end{array}$$

where S is substrate concentration; Q denotes for physiological state of the population, assumed to be proportional to the critical protein in a cell; X is biomass concentration of the microorganism; and A is LA concentration. In the first equation of the Equation (7), substrate utilization is modeled by biomass growth of the microorganism with growth yield coefficient $\gamma_{X/S}$ and the production of LA with yield coefficient $\gamma_{A/S}$.

### 2.5 The growth of *L*. *bulgaricus* in fermentation

**FIGURE 2:**
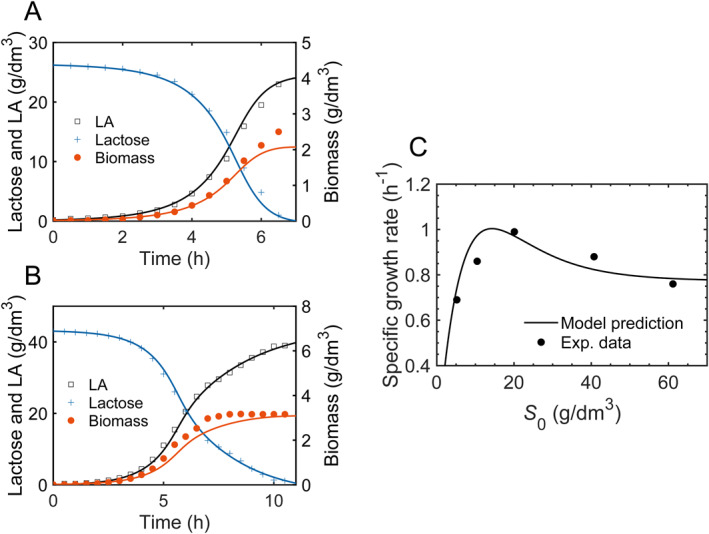
Model predictions of substrate inhibition. (A, B) Experimental and simulated growth profiles at two initial substrate concentrations of 26 g/dm^3^ (A), and 43 g/dm^3^ (B). All experimental data (+, substrate concentration, □, lactic acid concentration, ●, biomass concentration) are from the fermentation process of *L*. *bulgaricus* in Ref. [39] with copyright permission. (C) Model predictions of specific growth rates (solid line) over the range of initial substrate concentrations between $S_{0}$ = 2 g/dm^3^ to $S_{0}$ = 70 g/dm^3^. All parameters from the estimated parameters in Supporting Information S1: Table S1. No extra adjustable parameters were added to the predictions. Experimental data are from Ref. [39] with permission from the copyright holder.

To validate the model framework, we employ the published experimental observations of LA fermentation using *Lactobacillus bulgaricus* from lactose [39]. Starting from time course data of bacterial growth from initial lactose concentration $S_{0}$ = 26 g/dm^3^ and $S_{0}$ = 43 g/dm^3^ in the experiments, the model parameters are extracted using least squares fitting in terms of Equation (7). The parameters are set as $K_{S}$ = 8 g/dm^3^ and $Q_{0}=0.6$. The obtained parameters are presented in Supporting Information S1: Table S1. Consequently, numerical simulations of time profiles of substrate consumption, bacterial growth and LA production for various initial substrate concentrations can be simulated. For two cases of $S_{0}$ = 26 g/dm^3^ and $S_{0}$ = 43 g/dm^3^, the results are shown in Figure 2A,B.

In the next step, we numerically figure out specific growth rates for initial substrate concentrations $S_{0}$ in a wide range. As a result, the model predictions of specific growth rates between $S_{0}$ = 2 g/dm^3^ and $S_{0}$ = 70 g/dm^3^ are demonstrated in Figure 2C, with a critical initial substrate concentration of $S_{c}$ = 14.3 g/dm^3^ for maximum specific growth rate, which is consistent with experimental measurements of specific growth rates of *Lactobacillus bulgaricus* [39].

### 2.6 Microbial growth kinetics for different physiological states

To evaluate the lag phase behaviors, we investigate the effect of physiological states of the inoculum $Q_{0}$ on microbial growth dynamics for diverse substrate concentrations. Here we focus on two key properties of bacterial growth: the specific growth rate (μ) and the lag time (λ).

**FIGURE 3:**
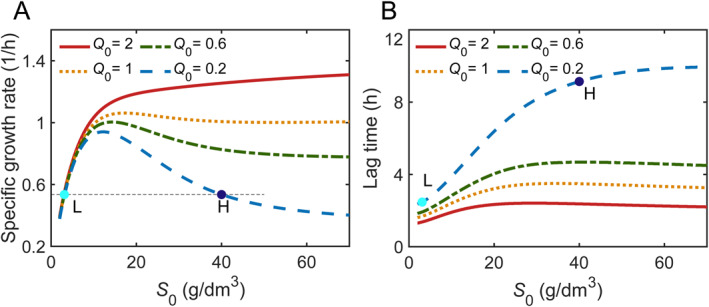
Microbial growth kinetics from different physiological states. The parameters come from *L*. *bulgaricus* as shown in Supporting Information S1: Table S1. (A) The specific growth rate as a function of initial substrate concentration for $Q_{0}$ = 0.2, 0.6, 1 and 2 (from bottom up). (B) The dependence of lag time on initial substrate concentration for $Q_{0}$ = 0.2, 0.6, 1 and 2 (from up bottom). The two dots “L” and “H” with the same growth rate are inoculated in low ($S_{0}$ = 3.1 g/dm^3^) and high ($S_{0}$ = 40 g/dm^3^) initial substrate concentrations, respectively.

Figure 3A shows the prediction results of dependence of specific growth rate on initial substrate concentration for various $Q_{0}$. Besides $Q_{0}$, other parameters are from Supporting Information S1: Table S1. An evident growth inhibition is observed for small values of $Q_{0}$ = 0.2 and 0.6, displaying a bell‐shaped curve of growth rate over a wide range of initial substrate concentrate. Remarkably, the growth inhibition gradually fades out with increasing $Q_{0}$, and an almost monotonically increasing curve of the growth rate relevant to initial substrate concentration was exhibited for larger values $Q_{0}$ = 1 and 2. In addition, for a given initial substrate concentration, the values of specific growth rate reduce with decreasing $Q_{0}$, especially at high initial substrate concentrations, demonstrating the typical substrate inhibition behavior.

Figure 3B displays a monotonically increasing lag time λ with initial substrate concentrations for diverse $Q_{0}$ values, implying that the cells need more time to take care of excess metabolism due to the overloading of substrate supply. The prolonged lag time with the increase of initial substrate concentration has been observed in microbial growth with substrate inhibition in the biological processes such as phenol biodegradation [11, 14], ethanol fermentation [41], LA production [42], and succinic acid production [43].

For different initial physiological state, it is shown that lag time λ is significantly prolonged with the reduced $Q_{0}$, especially at high initial substrate concentrations, indicating that those cells inoculated in the same environment but from a lower level of $Q_{0}$ need more time to adapt to exponential growth phase. Therefore, it can be inferred from the above that adequate preculture for improved $Q_{0}$ not only weakened the occurrence of substrate inhibition but shortened the lag phase in microbial growth as well. In fact, some previous studies have found that the preculture to late exponential phase contributed to minimize the lag phase in the main culture [39, 44].

Noticeably, the bell shaped growth rates for low level of $Q_{0}$ indicate that bacteria can grow at the same specific growth rates at low and high substrate concentrations respectively, such as two cases labeled by dots (“L” and “H”) in Figure 3A,B, yet those growing at high substrate concentration exhibit a much longer lag time as compared to cell growth in low substrate concentration.

### 2.7 The growth‐adaptation trade‐off and estimation of the maintenance energy

**FIGURE 4:**
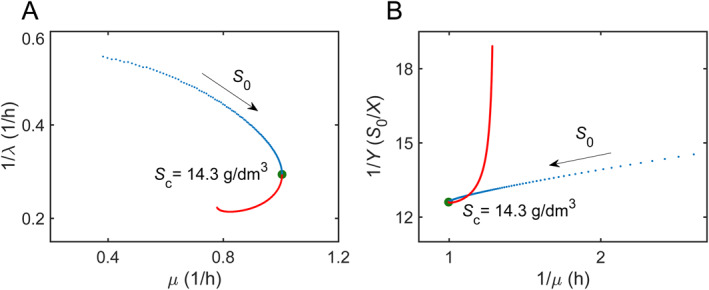
The simulated growth traits of *L*. *bulgaricus* growing on lactose from various initial substrate concentrations. Here $Q_{0}$ = 0.6, $X_{0}$ = 0.02 g/dm^3^. (A) A trade‐off strategy between the specific growth rate μ and adaptation 1/λ at low initial lactose concentrations (blue dots). The arrow mark points to the increase of initial substrate concentration. The critical concentration (dot) for maximum specific growth rate $S_{c}$ = 14.3 g/dm^3^. (B) Relationship between the inverse of growth yield 1/Y and the inverse of growth rate 1/μ of *L*. *bulgaricus* at various initial lactose concentrations. Parameters are from Supporting Information S1: Table S1.

To take advantage of nutrient in harsh environments, bacteria often regulate cellular metabolic activities to coordinate two and more growth traits, such as lag time λ, specific growth rate μ, and growth yield Y (biomass formed per amount of resource consumed) [24]. To determine the likely bacterial growth strategy in substrate inhibition, we numerically investigate the relation between the specific growth rate μ and the physiological adaptation 1/λ (denoted by the inverse of lag time) for various values of $S_{0}$. Figure 4A exhibits a trade‐off between the growth rate and the physiological adaptation under the conditions of lower substrate concentrations (blue regime) that is related to the allocation of limited energy resources. A previous experiment has observed a universal trade‐off between steady‐state growth rate and physiological adaptability of *Escherichia coli* in a nutrient fluctuating environment [45]. In contrast, the coupled suppression of specific growth rates (μ) and prolongation of lag phases (λ) can be a hallmark of substrate inhibition dynamics in high nutrient environments (red regime).

To better understand substrate utilization in microbial fermentation, we investigate the relationship of maintenance energy in substrate inhibition. Figure 4B plots the inverse of growth yield (1/Y) as a function of the inverse of specific growth rate (1/μ) in *Lactobacillus bulgaricus* fermentation using lactose. From the Pirt model, as shown in Equation (8) [46, 47, 48],

(8) $$\frac{1}{Y}=\frac{1}{Y_{G}}+\frac{m}{\mu }$$

where Y is the observed growth yield measuring mass of bacteria produced by per mass of substrate, and $Y_{G}$ is the theoretical growth yield measuring growth yield in the absence of maintenance costs, the slope of the 1/Y−1/μ relation in Figure 4B can be read as the maintenance energy coefficient m.

Figure 4B exhibits two distinctive relationships in terms of the slope of curves, separated by the critical initial substrate concentration $S_{c}$ = 14.3 g/dm^3^ at maximum growth rate. When initial substrate concentration is less than $S_{c}$, the maintenance energy coefficient m (the fitting slope of the blue regime) is in a relatively low level and almost a constant, indicating that more substrates are used for bacterial growth in this case. In contrast, when substrate concentration is larger than $S_{c}$, the maintenance energy coefficient m (the fitting slope of the red regime) becomes substrate dependent, increasing sharply with growing substrate concentrations. The emergence of substrate‐dependent maintenance energy has been observed across divergent microorganisms, such as *Chlamydomonas reinhardtii* growth on acetate, where substrate inhibition exacerbated the bioenergetic burden of microbial growth [47].

### 2.8 The growth of *L*. *casei* and LA productivity in fermentation

To test the universality of our model framework, we numerically investigate the fermentation process in Ref. [40], where Altıok et al. studied *Lactobacillus casei* fermentation in a batch culture from whey lactose within the initial substrate concentration range of 9.0–77.1 g/dm^3^. The model parameters (see Supporting Information S1: Table S1) are extracted by fitting Equation (7) to the experimental data of initial lactose concentration $S_{0}$ = 35.5 g/dm^3^ and $S_{0}$ = 48.1 g/dm^3^ in Ref. [40]. Using this set of parameters, the time courses of predictions of lactose consumption, biomass concentration of *L*. *casei* and LA production are calculated for various initial lactose concentrations. Supporting Information S1: Figures S1A,B,E,F illustrate that model predictions of fermentation process agree well with the experimental data, even beyond the range of two fitting cases of $S_{0}$ = 35.5 g/dm^3^ and $S_{0}$ = 48.1 g/dm^3^ (Figures S1C, D).

**FIGURE 5:**
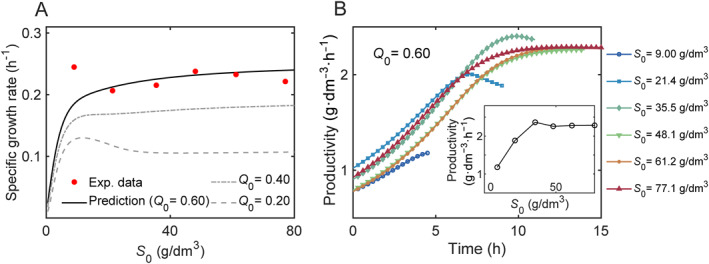
Effect of initial substrate concentration on the growth rates of *L*. *casei* and lactic acid productivity. (A) Model predictions of substrate dependent specific growth rate over a wide range of initial substrate concentrations. $Q_{0}$ = 0.20 (dash line), 0.40 (dash dot line), and 0.60 (solid line). The experimental data of specific growth rates (red dots) are calculated using data from Ref. [40] with copyright permission. (B) Model predictions of LA volume productivity as a function of time for experimental initial values of $S_{0}$ = 9.0, 21.4, 35.5, 48.1, 61.2, and 77.1 g/dm^3^ using Equation (9). Here, $Q_{0}$ = 0.60. The inset illustrates model predictions of productivity at the experimental cutoff time point for different initial substrate concentrations. See Supporting Information S1: Table S1 for the parameters.

To identify substrate inhibition in *L*. *casei* fermentation, we figure out the specific growth rates from log phase of the predicted bacterial growth profile within a lactose range of 0.5–80 g/dm^3^ for different $Q_{0}$, as shown in Figure 5A. Under the condition of experimental fermentation in Ref. [40], where $Q_{0}$ = 0.60, an monotonically increasing specific growth rate on initial substrate concentrations (solid line) can be observed. In this case, substrate inhibition is almost undetectable across all tested lactose concentrations, in consistent with the experimental results (red dots). When $Q_{0}$ is extended to a lower value of 0.20, a nonmonotonic substrate concentration‐dependent growth is obtained (dashed line), with a maximum specific growth rate at initial lactose of 17.1 g/dm^3^.

Batch fermentation is a commonly used method in industrial LA production but suffers productivity constraints from substrate and product inhibition. To find out the optimal volume productivity, we calculate the time‐dependent volume productivity for different initial substrate concentrations, using the ratio of the running product concentration A(t)−A(0) (g/dm^3^) to the running time point t (h) [49], as shown in Equation (9).

(9) $$\text{Productivity}=\frac{A\left(t\right)-A\left(0\right)}{t}$$

The model predictions of time evolution of LA productivity for $Q_{0}$ = 0.60 are presented in Figure 5B. All cases of productivity with initial lactose range of 9.0–77.1 g/dm^3^ achieve their maximum between 5 and 10 h. The highest productivity is around 2.4 g·dm^−3^·h^−1^ at around 10 h for initial lactose concentration of 35.5 g/dm^3^, in consistent with that in prior literature [40]. The inset in Figure 5B demonstrates the productivity values at the same fermentation time as in the experiments for those different $S_{0}$ [40]. At the end time of fermentation, the nutrients are almost exhausted except those of $S_{0}$ = 61.2 g/dm^3^ and $S_{0}$ = 77.1 g/dm^3^ (Supporting Information S1: Figure S1). The productivity values are similar and around 2.3 g/dm^3^ except for two lower initial substrate concentrations of 9.0 g/dm^3^ and 21.4 g/dm^3^, although their specific growth rates are approximately the same.

### 2.9 The growth of *L*. *plantarum* in fermentation and the effect of inoculum size

*Lactiplantibacillus plantarum* is one of the most commonly used LAB species that can utilize various sugars for LA production in fermentation industry [3]. A recent experiment [50] studied LA production by *L*. *plantarum* AC 11S from different initial lactose concentrations, and several models were introduced to describe the experimental data. It was concluded that the modified Gompertz equation was the best for solving only the equation for biomass growth, while a variant of the logistic equation for biomass growth was best for solving the entire set of differential equations for bacterial growth, substrate consumption, and product formation. The inconsistency in the model selection makes it difficult to evaluate the model effectiveness.

**FIGURE 6:**
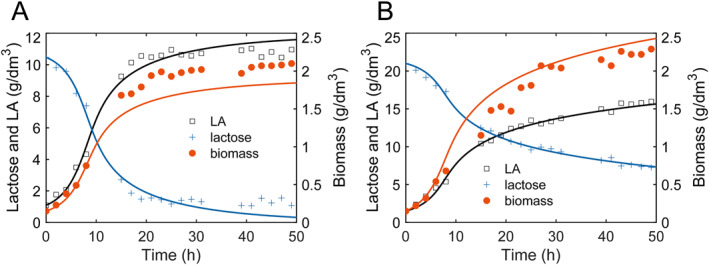
The simulation results (lines) and the experimental data of substrate consumption (+), biomass of *L*. *plantarum* (●), and lactic acid production (□) in the fermentation process using lactose at 11 g/dm^3^ (A) and 22 g/dm^3^ (B). The experimental data are from [50] with copyright permission. The parameters are in Supporting Information S1: Table S1.

Here, we aim at to understand the effect of substrate concentration and the inoculum size on *L*. *plantarum* fermentation in terms of the experimental observations from Ref. [50]. The model parameters are extracted by fitting the experimental data of $S_{0}$ = 11 g/dm^3^ and $S_{0}$ = 22 g/dm^3^ to Equation (7), as listed in Supporting Information S1: Table S1. Figure 6A,B show the simulation results of substrate consumption, biomass growth, and LA production for 11 g/dm^3^ and 22 g/dm^3^, respectively. The obtained parameter of β=0 indicates that LA production by *L*. *plantarum* fermentation is a growth‐associated process, which is consistent with the model results of Ref. [50].

**FIGURE 7:**
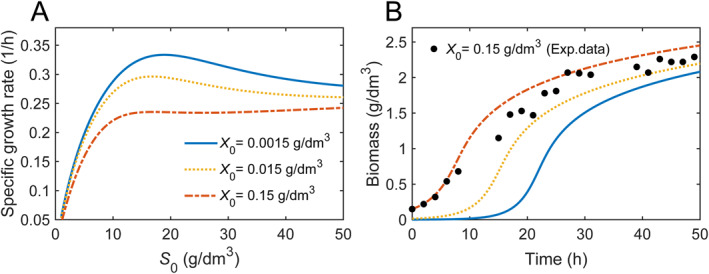
The influence of the inoculum size on bacterial growth. (A) The initial substrate concentration‐dependent specific growth rate inoculated from $X_{0}$ = 0.0015 g/dm^3^, $X_{0}$ = 0.015 g/dm^3^, and $X_{0}$ = 0.15 g/dm^3^ (from up bottom). (B) Temporal biomass growth profiles for different inoculum sizes of $X_{0}$ = 0.0015 g/dm^3^, $X_{0}$ = 0.015 g/dm^3^, and $X_{0}$ = 0.15 g/dm^3^ (from bottom up). The filled circles represented experimental data from Ref. [50] with copyright permission. The parameters are from Supporting Information S1: Table S1.

It has been known that cultivation conditions such as inoculum size are significantly crucial for microbial growth in batch culture, and thus the optimal LA production are greatly dependent on the selection of inoculum size [3, 51]. To study the relationship of inoculum size and the occurrence of substrate inhibition, we present the numerical simulations of the specific growth rates at three different inoculum concentrations of $X_{0}$ = 0.0015 g/dm^3^, $X_{0}$ = 0.015 g/dm^3^, and $X_{0}$ = 0.15 g/dm^3^ in *Lactiplantibacillus plantarum* fermentation (Figure 7A). The occurrence of substrate inhibition is observed evidently for the case of $X_{0}$ = 0.0015 g/dm^3^, and gradually weakened from $X_{0}$ = 0.015 g/dm^3^ to $X_{0}$ = 0.15 g/dm^3^. In addition, the growth rates reduce as the inoculum size increases, especially at the initial substrate concentrations where the maximum growth rate exists.

Figure 7B presents the predictions of time courses of biomass concentration for *X* _0_ = 0.0015, 0.015, and 0.15 g/dm^3^. Significantly, a higher level of inoculum size such as *X* _0_ = 0.15 g/dm^3^ is inclined to a shorter lag time, indicating high efficient fermentation process in this scenario. The measured experimental data (black dots) of biomass concentration in *Lactiplantibacillus plantarum* fermentation validated our prediction [50]. Therefore, the optimization of microbial growth can be related to the selection of cultivation conditions including an appropriate initial substrate concentration and an optimal inoculum size as well.

## 3 CONCLUSION AND DISCUSSION

In summary, we study substrate inhibition by an integrated two‐pathway model, taking into account a physiological adaptation process in response to the overloading of substrate supply. The model has captured a global view of bacterial growth dynamics including lag phase, exponential phase and stationary phase in batch fermentation over a wide range of substrate concentrations, using only one set of parameters. Consequently, the occurrence of substrate inhibition was able to be predicted in terms of bacterial growth profiles. This model approach has been validated across three LAB fermentation experiments available in the literature such as *L. bulgaricus*, *L. casei* and *L*. *plantarum* on lactose, demonstrating its universality beyond specific substrate–strain systems. It suggests that our integrative model is mathematically robust, relying on a minimal number of fitting parameters to predict the growth of bacterial species on carbon sources.

We have compared our model framework with other specific growth rate equations in characterizing substrate inhibition in batch fermentation kinetics, such as the modified Monod equations and the modified logistic equation in Refs. [39, 40, 49, 50]. Although the traditional methods can reproduce the experimental growth with substrate inhibition, microbial growth in those models was mostly considered to be related to the current environment, without taking into account cell history in preculture conditions. Our mechanistic model characterized the adaptation to a new medium of the cell growth dynamics as a combined effect of the external environment and the internal physiological state of the cells which is largely related to preculture conditions, enabling a preculture strategy to be a feasible approach to promote the yield and productivity in LA production.

Future research should continue the systematic experiments and model predictions of diverse microbial‐substrate combinations to evaluate model generality and predictive accuracy. Concurrently, the regulatory influence of crucial enzymes (e.g., *β*‐galactosidase activity) needs to be further investigated to quantitatively elucidate their role in substrate inhibition dynamics.

## 4 METHODS

### 4.1 Specific growth rate and lag time

In this work, the specific growth rate was calculated by the relationship of $\mu =\frac{1}{X}\frac{dX}{dt}=\frac{\ln\;X_{2}-\ln\;X_{1}}{t_{2}-t_{1}}$ in log phase, where X is the biomass concentration, and t is time. Specifically, the specific growth rate was calculated by the slope of the log phase in bacterial growth, and the time to reach the maximum value of the derivative of biomass was taken as lag time (Supporting Information S1: Figure S2), as was done in previously published techniques [15].

### 4.2 Parameter estimation

In this work, the kinetic model as shown in Equation (7) is used to describe the microbial fermentation process. There are 8 parameters ($\mu_{\max}$, $Q_{0}$, $K_{S}$, $K_{I}$, α, β, $\gamma_{X/S}$, $\gamma_{A/S}$) to be determined in these model equations. We make the parameter estimation using experimental data of substrate utilization, biomass growth and LA formation in *L*. *bulgaricus* [39], *L*. *casei* [40], and *L*. *plantarum* [50] fermentation process from published literature. The initial conditions of the model, as shown in Equation (7) read as: $S\left(t=0\right)=S_{0}$, $Q\left(t=0\right)=Q_{0}$, $X\left(t=0\right)=X_{0}$, and $A\left(t=0\right)=A_{0}$. It should be noted that the initial level of physiological state $Q_{0}$ is estimated by fitting unless specified otherwise, while the other initial values in the Equation (7) are assigned from the initial values in the experiment.

In this work, we employ an algorithm to simultaneously solve the model Equation (7) from multiple initial substrate concentrations. The sum of the squared differences (F) of the concentrations of biomass (X), substrate (S), and LA (A) between model predictions and experimental data of batch fermentations was minimized to identify the set of parameters. Here, the function F is formulated as following Equation (10),

(10) $$\begin{array}{l} F\left(\mu_{\max},Q_{0},K_{S},K_{I},\alpha ,\beta ,\gamma_{X/S},\gamma_{A/S}\right)\\ \begin{array}{cc} & = \end{array}\sum_{i=1}^{N}{\left(X\left(t_{i}\right)-X_{E}\left(t_{i}\right)\right)}^{2}+\sum_{i=1}^{N}{\left(A\left(t_{i}\right)-A_{E}\left(t_{i}\right)\right)}^{2}+\sum_{i=1}^{N}{\left(S\left(t_{i}\right)-S_{E}\left(t_{i}\right)\right)}^{2} \end{array}$$

where $t_{i}$ represents the time at which the i‐th group of experimental data was measured; $X\left(t_{i}\right)$, $A\left(t_{i}\right)$, and $S\left(t_{i}\right)$ are the simulated concentrations of biomass, LA, and substrate in terms of integrating the Equation (7); $X_{E}\left(t_{i}\right)$, $A_{E}\left(t_{i}\right)$, and $S_{E}\left(t_{i}\right)$ are the corresponding experimental data; N is the total number of experimental data.

The least‐squares fitting is performed to figure out the minimum of the function $F\left(\mu_{\max},Q_{0},K_{S},K_{I},\alpha ,\beta ,\gamma_{X/S},\gamma_{A/S}\right)$, using MATLAB’s lsqcurvefit function (Optimization Toolbox™, Version R2023b). Numerical integration of the equations are conducted via the forward Euler method. The fitting process terminates when the calculated parameter changes between iterations fall below the tolerance of $10^{-4}$.

In this work, Hill index is fixed at $n_{A}=5$, and $n_{Q}=2$. All the parameters estimated in this model are listed in Supporting Information S1: Table S1.

## AUTHOR CONTRIBUTIONS

**Guoxi Zheng**: Data curation; formal analysis; investigation; software; visualization; writing—review and editing. **Junwen Mao**: Conceptualization; data curation; formal analysis; funding acquisition; investigation; methodology; project administration; supervision; validation; writing—original draft; writing—review and editing.

## CONFLICT OF INTEREST STATEMENT

The authors declare no conflicts of interest.

## ETHICS STATEMENT

The authors declare that the study does not include animal and human experiments that violate ethics.

## CODE AVAILABILITY

The source codes are available on the GitHub website (Zheng‐GX/LAB_two‐pathway‐model.git).

## Supporting information

Supporting Information S1

## Data Availability

The data that support the findings of this study are available from the corresponding author upon reasonable request.

## References

[1] Abdel‐Rahman MA , Tashiro Y , Sonomoto K . Recent advances in lactic acid production by microbial fermentation processes. Biotechnol Adv. 2013;31(6):877–902. 23624242 10.1016/j.biotechadv.2013.04.002

[2] Abdel‐Rahman MA , Sonomoto K . Opportunities to overcome the current limitations and challenges for efficient microbial production of optically pure lactic acid. J Biotechnol. 2016;236:176–192. 27527396 10.1016/j.jbiotec.2016.08.008

[3] Rawoof SAA , Kumar PS , Vo DVN , Devaraj K , Mani Y , Devaraj T , et al. Production of optically pure lactic acid by microbial fermentation: a review. Environ Chem Lett. 2021;19(1):539–556.

[4] Ojo AO , de Smidt O . Lactic acid: a comprehensive review of production to purification. Processes. 2023;11(3):688.

[5] Mulchandani A , Luong JHT . Microbial inhibition kinetics revisited. Enzym Microb Technol. 1989;11(2):66–73.

[6] Tan Y , Wang Z‐X , Marshall KC . Modeling substrate inhibition of microbial growth. Biotechnol Bioeng. 1996;52(5):602–608. 18629933 10.1002/(SICI)1097-0290(19961205)52:5<602::AID-BIT7>3.0.CO;2-N

[7] Edwards VH . The influence of high substrate concentrations on microbial kinetics. Biotechnol Bioeng. 1970;12(5):679–712. 5489781 10.1002/bit.260120504

[8] Panikov NS . Kinetics, microbial growth. In: In encyclopedia of bioprocess technology: fermentation, biocatalysis and bioseparation. Nauka; 1991.

[9] Andrews JF . A mathematical model for the continuous culture of microorganisms utilizing inhibitory substrates. Biotechnol Bioeng. 1968;10(6):707–723.

[10] Kim DJ , Choi JW , Choi NC , Mahendran B , Lee CE . Modeling of growth kinetics for *Pseudomonas spp.* during benzene degradation. Appl Microbiol Biotechnol. 2005;69(4):456–462. 15856223 10.1007/s00253-005-1997-z

[11] Hill GA , Robinson CW . Substrate inhibition kinetics: phenol degradation by *Pseudomonas putida* . Biotechnol Bioeng. 1975;17(11):1599–1615.

[12] Panikov NS . Microbial growth kinetics: Chapman & Hall; 1995.

[13] Luong JHT . Generalization of monod kinetics for analysis of growth data with substrate inhibition. Biotechnol Bioeng. 1987;29(2):242–248. 18576382 10.1002/bit.260290215

[14] Goudar CT , Ganji SH , Pujar BG , Strevett KA . Substrate inhibition kinetics of phenol biodegradation. Water Environ Res. 2000;72(1):50–55.

[15] Adkar BV , Manhart M , Bhattacharyya S , Tian J , Musharbash M , Shakhnovich EI . Optimization of lag phase shapes the evolution of a bacterial enzyme. Nat Ecol Evolution. 2017;1(6):0149. 10.1038/s41559-017-0149 PMC5640271 28812634

[16] Himeoka Y , Kaneko K . Theory for transitions between exponential and stationary phases: universal laws for lag time. Phys Rev X. 2017;7(2):021049.

[17] Schultz D , Kishony R . Optimization and control in bacterial lag phase. BMC Biol. 2013;11:1–3. 24377387 10.1186/1741-7007-11-120 PMC3877865

[18] Lopatkin AJ , Collins JJ . Predictive biology: modelling, understanding and harnessing microbial complexity. Nat Rev Microbiol. 2020;18(9):507–520. 32472051 10.1038/s41579-020-0372-5

[19] Allen RJ , Waclaw B . Bacterial growth: a statistical physicist’s guide. Rep Prog Phys. 2018;82(1):016601. 30270850 10.1088/1361-6633/aae546 PMC6330087

[20] Bertrand RL . Lag phase is a dynamic, organized, adaptive, and evolvable period that prepares bacteria for cell division. J Bacteriol. 2019;201(7):697–718. 10.1128/JB.00697-18 PMC6416914 30642990

[21] Rolfe MD , Rice CJ , Lucchini S , Pin C , Thompson A , Cameron ADS , et al. Lag phase is a distinct growth phase that prepares bacteria for exponential growth and involves transient metal accumulation. J Bacteriol. 2012;194(3):686–701. 22139505 10.1128/JB.06112-11 PMC3264077

[22] Ginovart M , Carbó R , Portell X . Adaptation of *Saccharomyces* to high glucose concentrations and its impact on growth kinetics of alcoholic fermentations. Microorganisms. 2024;12(7):1449. 39065218 10.3390/microorganisms12071449 PMC11278885

[23] van der Pol EC , Eggink G , Weusthuis RA . Production of L(+)‐lactic acid from acid pretreated sugarcane bagasse using *Bacillus coagulans* DSM2314 in a simultaneous saccharification and fermentation strategy. Biotechnol Biofuels. 2016;9:1–12. 27872661 10.1186/s13068-016-0646-3 PMC5111225

[24] Brückner R , Titgemeyer F . Carbon catabolite repression in bacteria: choice of the carbon source and autoregulatory limitation of sugar utilization. FEMS Microbiol Lett. 2002;209(2):141–148. 12007797 10.1111/j.1574-6968.2002.tb11123.x

[25] Klumpp S , Hwa T . Bacterial growth: global effects on gene expression, growth feedback and proteome partition. Curr Opin Biotechnol. 2014;28:96–102. 24495512 10.1016/j.copbio.2014.01.001 PMC4111964

[26] Maier RM , Pepper IL . Bacterial growth. In: Environmental microbiology. 3th ed: Elsevier; 2015.

[27] Mao J , Blanchard AE , Lu T . Slow and steady wins the race: a bacterial exploitative competition strategy in fluctuating environments. ACS Synth Biol. 2015;4(3):240–248. 24635143 10.1021/sb4002008

[28] Venkatesh KV , Okos MR , Wankat PC . Kinetic model of growth and lactic acid production from lactose by *Lactobacillus bulgaricus* . Process Biochem. 1993;28(4):231–241.

[29] Nath A , Datta S , Chowdhury R , Bhattacharjee C . Fermentative production of intracellular β‐galactosidase by *Bacillus safensis* (*JUCHE 1*) growing on lactose and glucose—Modeling and experimental. Biocatal Agric Biotechnol. 2014;3(4):246–258.

[30] Xu Z , Li C , Ye Y , Wang T , Zhang S , Liu X . The β‐galactosidase LacLM plays the major role in lactose utilization of *Lactiplantibacillus plantarum* . LWT. 2022;153:112481.

[31] Hakkı Boyacı İ , Baş D , Ceyda Dudak F , Topçu A , Saldamlı İ , Özgür Şafak Şeker U , et al. Statistical modeling of β‐galactosidase inhibition during lactose hydrolysis. Food Biotechnol. 2006;20:79–91.

[32] Nath A , Mondal S , Chakraborty S , Bhattacharjee C , Chowdhury R . Production, purification, characterization, immobilization, and application of β‐galactosidase: a review. Asia Pac J Chem Eng. 2014;9(3):330–348.

[33] Zheleva P , Vasileva T , Mandadzhieva T , Ivanova I , Iliev I . Influence of lactose concentration on the α‐galactosidase and β‐galactosidase activity of *Lactobacillus plantarum* . Bulg. J. Agric. Sci. 2014;20:62–65.

[34] Monod J . The growth of bacterial cultures. Annu Rev Microbiol. 1949;3(1):371–394.

[35] Baranyi J , Roberts TA . A dynamic approach to predicting bacterial growth in food. Int J Food Microbiol. 1994;23(3‐4):277–294. 7873331 10.1016/0168-1605(94)90157-0

[36] Ram Y , Dellus‐Gur E , Bibi M , Karkare K , Obolski U , Feldman MW , et al. Predicting microbial growth in a mixed culture from growth curve data. Proc Natl Acad Sci USA. 2019;116(29):14698–14707. 31253703 10.1073/pnas.1902217116 PMC6642348

[37] Madar D , Dekel E , Bren A , Zimmer A , Porat Z , Alon U . Promoter activity dynamics in the lag phase of *Escherichia coli* . BMC Syst Biol. 2013;7:1–13. 24378036 10.1186/1752-0509-7-136 PMC3918108

[38] Dykhuizen DE , Dean AM , Hartl DL . Metabolic flux and fitness. Genetics. 1987;115(1):25–31. 3104135 10.1093/genetics/115.1.25 PMC1203059

[39] Burgos‐Rubio CN , Okos MR , Wankat PC . Kinetic study of the conversion of different substrates to lactic acid using *Lactobacillus bulgaricus* . Biotechnol Prog. 2000;16(3):305–314. 10835228 10.1021/bp000022p

[40] Altıok D , Tokatlı F , Harsa Ş . Kinetic modelling of lactic acid production from whey by *Lactobacillus casei* (NRRLB‐441). J Chem Tech Biotechnol. 2006;81(7):1190–1197.

[41] Thatipamala R , Rohani S , Hill GA . Effects of high product and substrate inhibitions on the kinetics and biomass and product yields during ethanol batch fermentation. Biotechnol Bioeng. 1992;40(2):289–297. 18601115 10.1002/bit.260400213

[42] Fu W , Mathews AP . Lactic acid production from lactose by *Lactobacillus plantarum*: kinetic model and effects of pH, substrate, and oxygen. Biochem Eng J. 1999;3:163–170.

[43] Lin SKC , Du C , Koutinas A , Wang R , Webb C . Substrate and product inhibition kinetics in succinic acid production by actinobacillus succinogenes. Biochem Eng J. 2008;41(2):128–135.

[44] Lorántfy B , Johanson A , Faria‐Oliveira F , Franzén CJ , Mapelli V , Olsson L . Presence of galactose in precultures induces lacS and leads to short lag phase in lactose‐grown *Lactococcus lactis* cultures. J Ind Microbiol Biotechnol. 2019;46(1):33–43. 30413923 10.1007/s10295-018-2099-0 PMC6339885

[45] Basan M , Honda T , Christodoulou D , Hörl M , Chang Y‐F , Leoncini E , et al. A universal trade‐off between growth and lag in fluctuating environments. Nature. 2020;584(7821):470–474. 32669712 10.1038/s41586-020-2505-4 PMC7442741

[46] Pirt SJ . Maintenance energy: a general model for energy‐limited and energy‐sufficient growth. Arch Microbiol. 1982;133(4):300–302. 7171288 10.1007/BF00521294

[47] Chen F , Johns MR . Relationship between substrate inhibition and maintenance energy of *Chlamydomonas reinhardtii* in heterotrophic culture. J Appl Phycol. 1996;8(1):15–19.

[48] Berrios J , Theron CW , Steels S , Ponce B , Velastegui E , Bustos C , et al. Role of dissimilative pathway of *Komagataella phaffii* (*Pichia pastoris*): formaldehyde toxicity and energy metabolism. Microorganisms. 2022;10(7):1466. 35889185 10.3390/microorganisms10071466 PMC9321669

[49] Gordeeva YL , Rudakovskaya EG , Gordeeva EL , Borodkin AG . Mathematical modeling of biotechnological process of lactic acid production by batch fermentation: a review. Theor Found Chem Eng. 2017;51(3):282–298.

[50] Popova‐Krumova P , Danova S , Atanasova N , Yankov D . Lactic acid production by *Lactiplantibacillus plantarum* AC 11S—kinetics and modeling. Microorganisms. 2024;12(4):739. 38674683 10.3390/microorganisms12040739 PMC11051871

[51] Alvarez MM , Aguirre‐Ezkauriatza EJ , Ramírez‐Medrano A , Rodríguez‐Sánchez Á . Kinetic analysis and mathematical modeling of growth and lactic acid production of *Lactobacillus casei* var. *rhamnosus* in milk whey. J Dairy Sci. 2010;93(12):5552–5560. 21094727 10.3168/jds.2010-3116

