# Supplementary material for: Mechanistic two‐pathway modeling of substrate inhibition in lactic acid bacteria for enhanced fermentation control
Source: Quant Biol. 2025 Oct 6;14(1):e70019. doi: 10.1002/qub2.70019 (PMC12806044; doi:10.1002/qub2.70019)
Supplement: Supplementary file 1 — Supporting Information S1 [file QUB2-14-e70019-s001.docx]

**Supporting Information**

**for**

**Mechanistic two-pathway modeling of substrate inhibition in lactic acid bacteria for enhanced fermentation control**

Guoxi Zheng, Junwen Mao

**Contents**

[1 Parameters 2](#_Toc78235105)

[2 Figures 3](#_Toc78235106)

# 1 Parameters

Table S1. Estimated parameter values in this work.

| Parameter | Description [Unit] | Parameter values | | |
| --- | --- | --- | --- | --- |
|  |  | *L. bulgaricus*^1^ | *L. casei*^2^ | *L. plantarum*^3^ |
| *$\mu_{\max}$* | maximum specific growth rate [1/h] | 2.17 | 0.68 | 0.58 |
| *$K_{S}$* | Monod constant [g/dm^3^] | 8* | 4.89 | 9.54 |
| *$K_{I}$* | product inhibition constant [g/dm^3^] | 21.07 | 23.48 | 7.11 |
| ** | substrate inhibition constant [g/dm^3^] | ** | ** | ** |
| $\alpha$ | growth-associated product coefficient [g lactic acid/g biomass] | 10.56 | 2.37 | 6.20 |
| *$\beta$* | non-growth-associated product coefficient [g lactic acid/g biomass h] | 0.46 | 0.28 | 0 |
| *$\gamma_{X/S}$* | growth yield coefficient [g biomass/g substrate] | 0.34 | 3.38×10^4^ | 7.93 |
| *$\gamma_{A/S}$* | product yield coefficient [g lactic acid/g substrate] | 1.19 | 0.70 | 1.06 |
| *$v$* | rate of adjustment to the new growth environment [1/h] | *$\nu=\mu_{\max}$* | *$\nu=\mu_{\max}$* | *$\nu=\mu_{\max}$* |
| *$Q_{0}$* | initial level of physiological state of the population | 0.6* | 0.60 | 0.94 |
| *$n_{A}$* | coefficient of product inhibition | 5* | 5* | 5* |
| *$n_{Q}$* | coefficient of substrate inhibition | 2* | 2* | 2* |

^*^In this work, they are fixed parameters.

^1^The fermentation substrate was lactose.

^2^The fermentation substrate was whey lactose.

^3^The fermentation substrate was lactose.

# 2 Figures

| 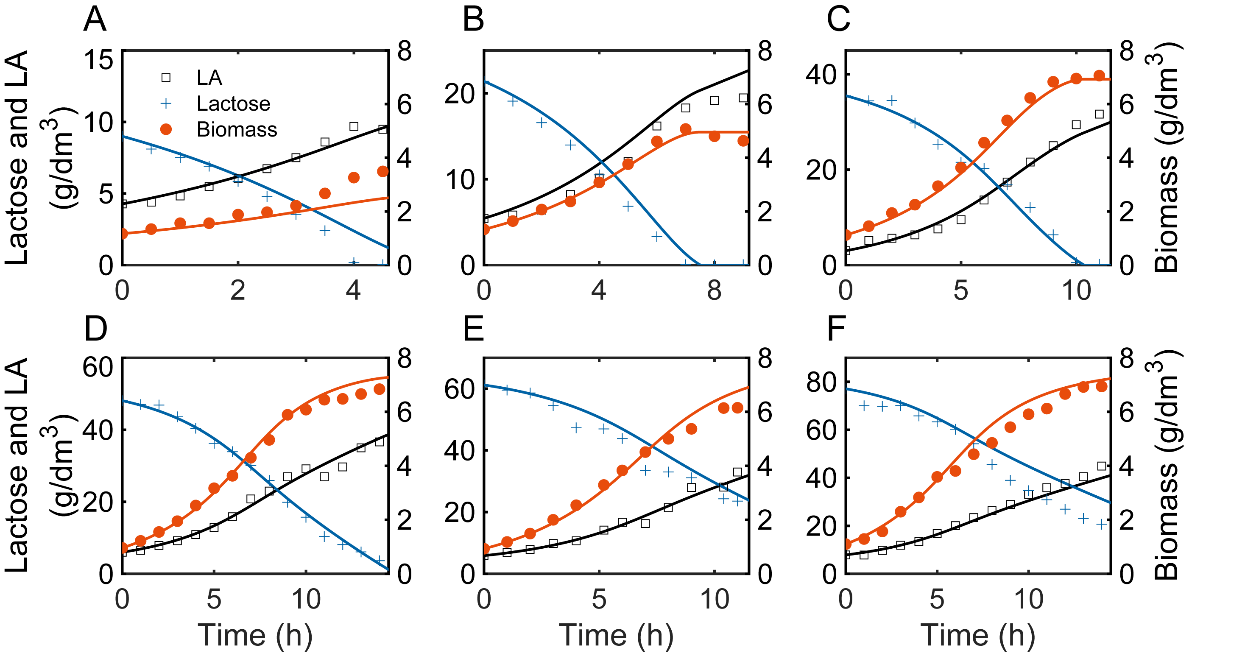 |
| --- |
| **Figure S1**. Fermentation kinetics of *L.casei* for different initial substrate concentrations. The concentrations are (A) 9.0 g/dm^3^, (B) 21.4 g/dm^3^, (C) 35.5 g/dm^3^, (D) 48.1 g/dm^3^, (E) 61.2 g/dm^3^, (F) 77.1 g/dm^3^ respectively. Experimental data (+, substrate concentration, □, lactic acid concentration, ●, biomass concentration) are from the study of Altiok et al. [1]. (C) and (D) present the fitted results, while (A), (B), (E), and (F) are predicted results. (A-F) share the same set of parameters. |

| 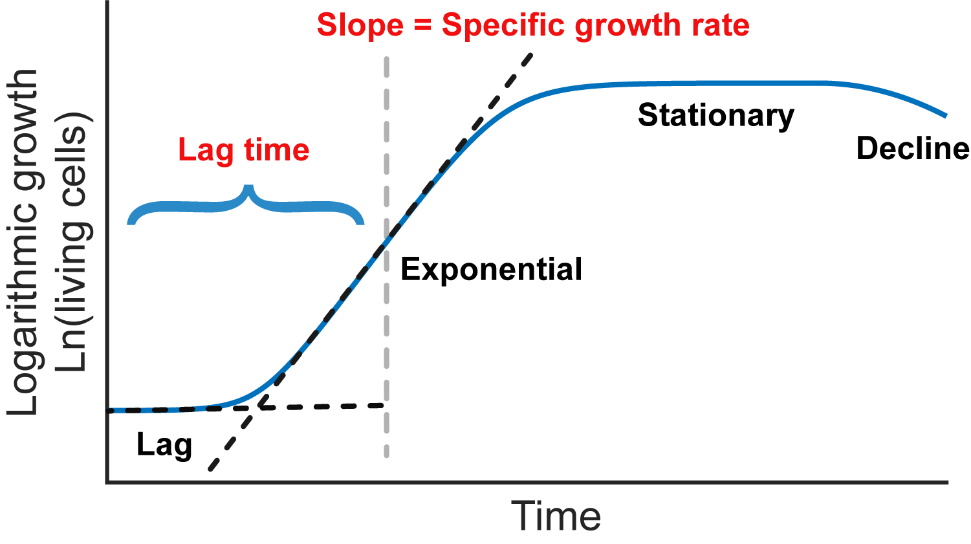 |
| --- |
| **Figure S2**. Schematic of estimation of lag time and growth rate. The specific growth rate in this paper is defined as the slope of microbial exponential phase growth under semi-logarithmic coordinates. The lag time in this study is defined as the time when the specific growth rate reaches its maximum value [2]. |

**REFEREENCES**

1. Altıok, D., Tokatlı, F., Harsa, Ş. Kinetic modelling of lactic acid production from whey by *Lactobacillus casei* (NRRLB-441). *J. Chem. Technol. Biot.* **2006**, *81*, 1190-1197.
2. Adkar, B.V., Manhart, M., Bhattacharyya, S., Tian, J., Musharbash, M., Shakhnovich, E.I. Optimization of lag phase shapes the evolution of a bacterial enzyme. *Nat. Ecol. Evolution.* **2017**, *1*, 0149.
